# Exogenous application of melatonin to mitigate drought stress-induced oxidative damage in *Phoebe sheareri* seedlings

**DOI:** 10.7717/peerj.15159

**Published:** 2023-04-17

**Authors:** Guifang Li, Yanzhen Li, Yuzi Zhu, Wenjun Zheng, Mengxi Li, Jinlong Hu, Yongjun Fei, Sijia Zhu

**Affiliations:** 1 College of Tourism & Landscape Architecture, Guilin University of Technology/College of Plant and Ecological Engineering, Guilin, China; 2 College of Horticulture and Gardening, Yangtze University, Jingzhou, China

**Keywords:** *Phoebe sheareri*, Melatonin, Drought stress, Photosynthesis, Antioxidant defense

## Abstract

**Background:**

Drought stress is a major prevalent environmental factor impairing growth. Melatonin mitigates the impacts of drought stress on plants. However, melatonin’s role in *Phoebe sheareri (Hemsl.) Gamble* (*P. sheareri*) is unknown. We aimed to reveal the protective effects of melatonin on *P. sheareri* seedlings under drought conditions.

**Methods:**

Melatonin was sprayed under drought or normal water conditions. The parameters, including growth, physiological factors, and phytohormones of *P. sheareri*, were examined.

**Results:**

Compared to the normal control group, drought stress inhibited the growth of seedlings and significantly reduced the content of carotenoids, SOD, POD, APX, PPO, CAT, GR, and soluble sugars, and increased the contents of MDA, O_2_^•−^, proline, soluble proteins, ABA, and JA-Me in *P. sheareri* seedlings. However, melatonin treatment significantly reversed the adverse drought-induced responses and promoted the *P. sheareri* seedling’s growth. Moreover, the heatmap and principal component analysis suggested a high similarity in the behavior patterns of the six measured antioxidant enzymes in *P. sheareri* seedlings.

**Conclusion:**

Our study reported for the first time that melatonin has a protective role in *P. sheareri* seedlings under drought-stress conditions. This role is related to ROS scavenging, activation of antioxidant enzymes, and crosstalk of phytohormones. This study provided a theoretical basis for improving the ability of *P. sheareri* adapted to arid environments.

## Introduction

*Phoebe sheareri* (Hemsl.) Gamble (*P*. *sheareri*) is a valuable and an endangered species in China’s National Class II protected wild plants. *P*. *sheareri* belongs to the genus *Phoebe* of the family Lauraceae. *Phoebe* mainly refers to the wood of the genus *Phoebe nees*, including *Phoebe puwenensis* W. C. Cheng and *Phoebe microphylla* H. W. Li, and *Machilus nees*, including *P*. *sheareri*, *Machilus zuihoensis*, and shuinan (Ding et al., 2015). Among them, *P. sheareri* is widely known for its hardwood, straight texture, and better aesthetic appearance, making it an ideal material for solid wood furniture and an excellent landscaping tree species. Currently, *P*. *sheareri* is an endemic species in China, mainly distributed in the evergreen broad-leaved forests below 1,000 m above sea level in the south of the Yangtze River Basin, such as Hubei province (He et al., 2018). However, due to weak natural regeneration ability and artificial deforestation, the wild *P. sheareri* resources have almost depleted (Wang et al., 2020). Therefore, understanding the adverse effects of environmental stresses on *P. sheareri* is of great significance for protecting *P*. *sheareri* resources.

Drought stress is the most prevalent environmental factor limiting plant growth and natural regeneration. At the cellular level, the damage caused by drought stress to plants is reflected by changing the plants’ metabolic activity and cell biological functions and limiting plants’ growth and development (Mahmood et al., 2019). The oxidative damage caused by the accumulation of reactive oxygen species (ROS), hydroxyl radical, H_2_O_2_, superoxide radical [O_2_^•−^], and singlet oxygen [^1^O_2_]), the major factors for drought stress, affect plant growth by injuring the integrity of the cell membrane by accelerating lipid peroxidation (Jain et al., 2019; Mohi-Ud-Din et al., 2021). Notably, plants have evolved multiple mechanisms of antioxidant systems to defend against or mitigate the toxicity of ROS. For example, plants can effectively scavenge ROS via different antioxidant enzyme components, including peroxidase (POD), catalase (CAT), superoxide dismutase (SOD), glutathione reductase (GR), polyphenol oxidase (PPO), ascorbate peroxidase (APX) (Mohi-Ud-Din et al., 2021), and non-enzymatic antioxidants components, including carotenoids, glutathione, and others (Gill & Tuteja, 2010). In recent years, with climate change, more frequent and severe drought in Hubei (Zheng & Liu, 2020) has become a significant risk factor for the survival and growth of *Phoebe* species. Although, some studies have reported the effects of drought stress on *Phoebe* species and proposed some drought resistance measures, such as *Phoebe zhennan* (Tariq et al., 2017) and *Phoebe hunanensis* (Yang et al., 2022). For example, Hu et al. (2015) found that the antioxidant defense system and osmolytes are essential in *Phoebe zhennan* S. Lee during recovery after drought stress. Tariq et al. (2019) proved that nitrogen supplementation could alleviate the growth and metabolism impairments induced by drought in *Phoebe zhennan*. However, research on drought resistance in *P*. *sheareri* still needs to be completed.

Melatonin is a hormone synthesized with serotonin as a substrate and secreted by the pineal gland. It can be found in several animals, plants, and bacteria that contain the pineal gland (Hardeland, Pandi-Perumal & Cardinali, 2006). Although the major understanding of melatonin in the past has been limited to chronobiotic (Arendt & Skene, 2005), more and more studies have demonstrated that melatonin has a protective role in plant oxidative stress as a plant growth regulator and/or biostimulator (Arnao & Hernandez-Ruiz, 2014). For example, endogenous triggering and exogenous administration of melatonin remodels redox homeostasis and enhances plant tolerance to cadmium by boosting the antioxidant defense system (Gu et al., 2021), possibly for other heavy metals (Hoque et al., 2021). In addition, melatonin has the ability to alleviate plant stress from common abiotic soil factors, such as salinity, alkalinity, and acidity (Moustafa-Farag et al., 2020). Importantly, melatonin has shown protection from drought stress, such as soybean (Imran et al., 2021) and maize (Ahmad et al., 2019). For example, under drought stress, long-term application of melatonin can promote the growth of apples by improving nutrient absorption (Liang et al., 2018) and exert a defensive role in maize by boosting the efficiency of photosystem II photochemistry (Fleta-Soriano et al., 2017). Sharma & Zheng (2019) systematically demonstrated the protective effects of melatonin at the physiological and molecular levels. However, whether melatonin can improve the tolerance of *P*. *sheareri* to drought stress has yet to be reported.

In the present study, we aimed to reveal the effects of drought stress on *P*. *sheareri* seedlings and whether melatonin can protect plants against drought. The mechanism by which melatonin protects plants from drought stress will be discussed superficially. This study provided evidence for *P*. *sheareri* protection after applying melatonin and a new strategy to improve *P*. *sheareri* seedlings’ survival under drought conditions.

## Materials & Methods

### Plant material and experimental design

A pot experiment was performed from August 2020 to February 2021 under natural light at the west campus of Yangtze University, Jingzhou, China (111°15′∼114°05′E, 29°26′∼31°37′N). The seeds of *P. sheareri* were collected and stored in the laboratory yearly. The *P. sheareri* seeds were buried in sandy soil, growing naturally without special treatment to harvest the annual seedlings. One-year-old healthy *P*. *sheareri* seedlings were used in this experiment, and one *P*. *sheareri* seedling was planted per pot. The cultivating matrix consisted of nutritive soil, river sand, and perlite in 1:1:1. *P*. *sheareri* seedlings were grouped into four groups. Each group was randomly assigned 30 seedlings: control check (CK) group (*n* = 30), drought stress (D) group (*n* = 30), drought stress + melatonin treatment (MD) group (*n* = 30), and melatonin treatment (M) group (*n* = 30). After three months of routine management, the experimental treatment was started in November. Each group of plants underwent the following treatments:

For the CK group, the plants underwent normal water (Hoagland nutrient solution) treatment. For the D group, during drought stress, the watering of plants was stopped until the soil moisture content was reduced to 0%. For the MD group, during drought stress, the watering of plants was stopped until the soil moisture content was reduced to 0%. Then, 100 µmol/L of melatonin was sprayed on the foliar surface simultaneously. Once every four days, a total of five treatments were administered. For the M group, the plants underwent normal water (Hoagland nutrient solution) treatment and foliar sprayed with 100 µmol/L of melatonin once every four days. The leaves of 30 seedlings in each group were collected at the end of the experiment, mixed, and randomly divided into three parts for subsequent detection of physiological parameters.

### Measurement of carotenoids (Car)

According to the product instructions, the Car content was evaluated with Plant Carotenoid Content Detection Kit (KLP1046, KangLang Bio. China) according to the product instructions. Briefly, Reagent I and Reagent II were mixed in 2:1 to obtain the extraction solution. The leaf samples were dried at 60 °C and crushed, then passed through a 100-mesh sieve. The sample (0.01 g) was dissolved in 2 mL extract solution and shaken at 37 °C for 60 min. Finally, the absorption values of blank and sample tubes were measured at 440 nm. Car content = 1.6×(OD_sample_ −OD_blank_)/sample weight.

### Activities of antioxidant enzymes

The SOD activity was measured by using Superoxide Dismutase (SOD) Test Kit (NBT method) (JN24888, Jining Shiye, China). Briefly, the leaf sample and extraction solution were mixed in 1:10 (g: mL) and shaken at 4 °C for 30 min. The samples were centrifuged at 4 °C for 10 min at 8,000 g, and the supernatant was tested. This was followed by the addition of 180 µL of supernatant, 45 µL of Reagent I, 2 µL of Reagent II, 35 µL of Reagent III, and 100 µL of Reagent IV into tubes, mixed and incubated for 30 min at 25 °C. Finally, the OD value was measured at 560 nm. SOD activity = 11.11× percentage inhibition/(1 − percentage inhibition)/Cpr. Cpr means the protein concentration of tissue homogenate.

The CAT activity was measured by the Catalase Test (CAT) Kit (ultraviolet absorption method) (JN24729, Jining Shiye, China). Briefly, the leaf sample and normal saline were mixed in 1:9 (g: mL) and shaken at 4 °C. The samples were centrifuged at 25 °C for 10 min at 2,500 g, and the supernatant was tested. This was followed by adding 0.05 mL of supernatant, 1 mL of Reagent I, and 0.1 mL of Reagent II, and placed in water maintained at 37 °C for 60 min. Afterward, 1 mL of Reagent III and 0.1 mL of Reagent IV were added. Finally, the OD values of samples were measured at 405 nm. CAT activity = (OD_blank_ –OD_sample_) × 271 × [1 / (60 × 0.05)]/Cpr. Cpr means the protein concentration of tissue homogenate.

The POD activity was measured by the Peroxidase (POD) Test Kit (spectrophotometric method) (JN24754, Jining Shiye, China). Briefly, the leaf samples and extraction solution were mixed in 1:10 (g:mL) and shaken at 4 °C. The samples were centrifuged at 4 °C for 10 min at 8,000 g, and the supernatant was tested. The absorption value of POD was detected at 470 nm using a spectrophotometer. This was followed by adding 15 µL of supernatant, 270 µL of double distilled water, 520 µL of Reagent I, 130 µL of Reagent II, and 135 µL of Reagent III into 1 mL of the cuvette, quickly mixed, and then the OD values A1 and A2 at 30 s and 90 s were measured. ΔA = A1 − A2. POD activity = 7133  × ΔA/sample weight.

The APX activity was measured by the Ascorbate peroxidase (APX) Test Kit (A123-1-1, Nanjing Jiancheng Bioengineering Institute, China). Briefly, the leaf samples were dried at 60 °C and crushed, then passed through a 100-mesh sieve. The sample (0.1 g) was dissolved in 1 mL of Reagent I and shaken at 4 °C for 60 min. The samples were centrifuged at 4 °C for 20 min at 13,000 g, and the supernatant was tested. The absorption value of APX was detected at 290 nm using a spectrophotometer. This was followed by adding 100 µL of supernatant, 700 µL of preheated Reagent I, 100 µL of Reagent II, and 100 µL of Reagent III into 1 mL of quartz cuvette and quickly mixed. The OD values A1 and A2 at 10 s and 130 s were measured. ΔA = A1 − A2. APX activity = 1786  × ΔA/sample weight.

The PPO activity was measured by the Polyphenol Oxidase (PPO) Test Kit (Colorimetric method) (A136-1-1, Nanjing Jiancheng Bioengineering Institute, China). Briefly, the leaf samples and Reagent I extraction solution were mixed in 1:10 (g:mL) and shaken at 4 °C for 30 min. The samples were centrifuged at 25 °C for 10 min at 8,000 g, and the supernatant was tested. This was followed by adding 150 µL of supernatant, 600 µL of Reagent II, and 150 µL of Reagent III in 1 cm of the cuvette, mixed and incubated for 10 min at 37 °C, followed by 5 min in a boiling water bath. Finally, the OD values of the samples were measured at 420 nm. PPO activity = (OD_sample_ − OD_blank_)/0.01× (V_ReagentI_/sample weight) × (V_total_/0.15 mL) × (1/V_total_) × 0.1 min.

The GR activity was measured by the Glutathione Reductases (GR) assay kit (Ultraviolet colorimetric method) (A062-1-1, Nanjing Jiancheng Bioengineering Institute, China). Briefly, the leaf samples and normal saline were mixed in 1:9 (g:mL) and shaken at 4 °C for 30 min. The samples were centrifuged at 25 °C for 10 min at 2,500 g, and the supernatant was tested. This was followed by adding 20 µL of supernatant into 2.4 mL solution and mixed quickly. After 30 s of reaction, the OD value at 340 nm was measured in a 1 cm quartz cuvette and named A1. Next, the mixed solution was bathed at 37 °C for 2 min, followed by the measurement for 2.5 min of OD value, named A2. GR activity = [(A1 − A2)/6.22]/2 min/(V_sample_ × Cpr) × N. Cpr means the protein concentration of tissue homogenate, and N means the dilution ratio of the sample.

### Oxidative stress indicators of malondialdehyde (MDA) and O_2_^•−^

The MDA content was measured with the Plant Malondialdehyde (MDA) assay kit (Colorimetric method) (A003-3-1, Nanjing Jiancheng Bioengineering Institute, China). Briefly, the leaf samples and Reagent V extraction solution were mixed in 1:9 (g:mL) and shaken at 4 °C for 10 min. The samples were centrifuged at 25 °C for 10 min at 4,000 g, and the supernatant was tested. This was followed by adding 50 µL of supernatant and 1 mL of working solution. The OD value at 530 nm was measured after boiling in a water bath for 20 min. The MDA content = [(OD_sample_ − OD_blank_)/(OD_standard sample_ − OD_blank_)] ×10 nmol/mL/(sample weight/V_total_).

The O_2_^•−^ content was measured by oxygen free radical test kit/colorimetric method (ml094977, Shanghai Enzyme-linked Biotechnology, China). Around 1 g of leaves were weighed, 2 mL of pre-cooled O2-LysisBuffer was added, and homogenized under the ice-bath condition. Next, the samples were centrifuged at 10,000 g at 4 °C for 1 min, and the supernatant was collected. After the NO_2_-standard was restored to room temperature, it was diluted in different multiples to construct the standard curve. This was followed by adding 0.25 mL of supernatant, 0.25 mL of O_2_-LysisBuffer, and 0.5 mL of hydroxylamine solution and placed in water maintained at 25 °C for 20 min. Afterward, 0.5 mL of aminobenzene sulfonic acid chromogenic solution and 0.5 mL of naphthylamine chromogenic solution were added. Finally, the OD values of the samples were measured at 530 nm after placing them in a water bath at 30 °C for 30 min. The O_2_^•−^ content was calculated according to the standard curve.

### Quantitation of soluble protein (SP), soluble sugar (SS), and proline (Pro) content

The SP content was tested by Bradford method according to a previous study (Abdel Latef & Tran, 2016). The SS content was detected by the plant soluble sugar content test kit (Colorimetric method) (A145-1-1, Nanjing Jiancheng Bioengineering Institute, China). Briefly, the leaf samples and double distilled water were mixed in 1:10 (g: mL), shaken, and placed in boiling water for 10 min. The samples were centrifuged at 25 °C for 10 min at 4,000 g, and the supernatant was tested. This was followed by adding 200 µL of supernatant, 100 µL of working solution, and 1 mL of concentrated sulfuric acid. The OD value at 620 nm was measured after placing the samples in a boiling water bath for 10 min. SP content = [(OD_sample_ − OD_blank_)/(OD_standard sample_ − OD_blank_)] ×100 µg/mL/(sample weight/V_total_) × N. N means the dilution ratio of the sample.

The content of Pro was detected by Proline Assay Test Kit (Colorimetric method) (A107-1-1, Nanjing Jiancheng Bioengineering Institute, China), according to the product instructions. Briefly, the leaf samples and Reagent I extraction solution were mixed in 1:9 (g:mL) and shaken at 4 °C. The samples were centrifuged at 25 °C for 10 min at 3,500 g, and the supernatant was tested. This was followed by adding 0.5 mL of supernatant, 1 mL of Reagent II, and 1 mL of Reagent III. The OD value at 520 nm was measured after placing the samples in a boiling water bath for 30 min. Pro content = [(OD_sample_ − OD_blank_)/(OD_standard sample_ –OD_blank_)] ×5 µg/mL/(sample weight/V_total_) × N. N means the dilution ratio of the sample.

### Endogenous phytohormones detection

The extraction and content quantification of endogenous jasmonic acid methyl ester (JA-Me) and abscisic acid (ABA) were performed as described previously (Imran et al., 2021; Li et al., 2018). Endogenous phytohormones were detected by enzyme-linked immunosorbent assays kits that the Laboratory of Molecular Plant Breeding of Yangtze University supplied.

### Drought resistance coefficient

The drought resistance coefficient (DRC) of 14 single indicators involved in this study was calculated using the following formula: DRC = *I*_*d*_/ *I*_*CK*_.

In the formula, *I*_*d*_ and *I*_*M*_ are the measured values of a certain index in the treatment and CK groups, respectively. The comprehensive coefficient of drought resistance was calculated using the following formula: Comprehensive coefficient of drought resistance = $ \frac{1}{n} {\mathop{\sum }\nolimits }_{i=1}^{n}DRC$.

### Statistical analysis

After all the data passed the normality test of Kolmogorov–Smirnov, Brown-Forsythe test, and Bartlett’s tests were used to analyze the homogeneity of variance of the data. When the data satisfied the homogeneity of variance, one-way ANOVA following Tukey’s multiple comparisons test was conducted. Otherwise, Dunnett’s multiple comparisons test was used. The parameter contained a 95% confidence interval. The significance cutoff of the *p*-value was 0.05. The data were visualized by Graphpad Prism 9.0. Principal component analysis (PCA) and heatmap were analyzed by Sangerbox (http://www.sangerbox.com) and R v.4.1.0 for Windows (http://CRAN.R-project.org/).

## Results

### Melatonin supported plant growth and carotenoid content

**Figure 1:**
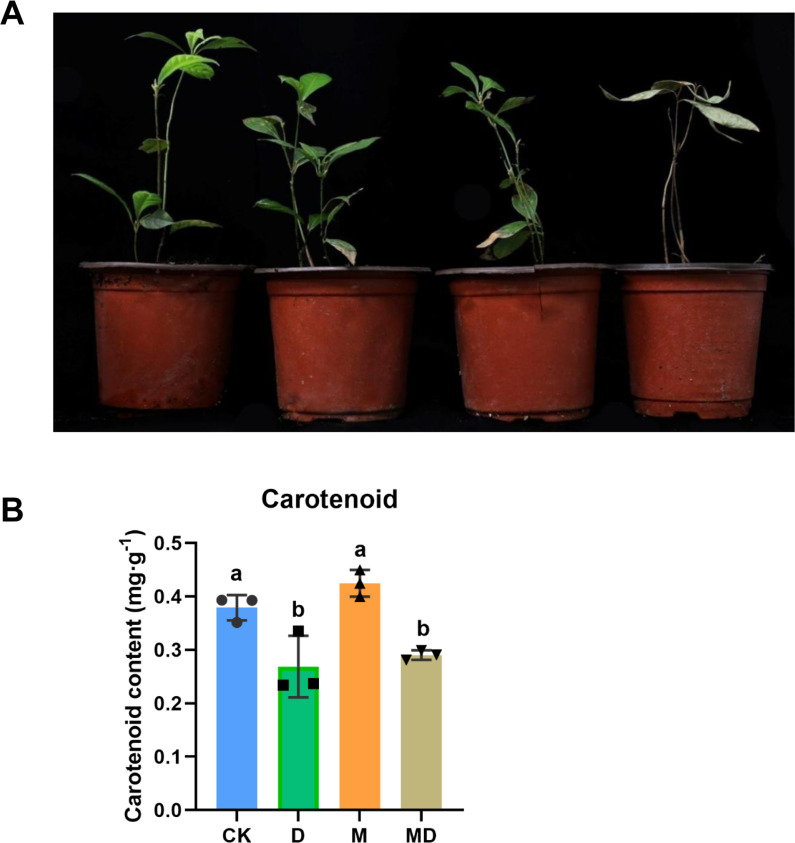
Melatonin supported plant growth and carotenoidcontent. (A) The morphology observation of *P*. *sheareri* seedlings in four groups. (B) The carotenoid content in *P*. *sheareri* seedlings in four groups.

We first observed the appearance of *P*. *sheareri* seedlings in four groups in drought conditions (Fig. 1A). In the CK and M groups, the seedlings did not experience drought stress, and the plants grew upright with bright green leaves and sprouted new leaves. However, the phenotypic morphology of *P*. *sheareri* seedlings in the D group exhibited that all leaves were yellowish, wilted and drooped, curled, and dried with severe dehydration. The leaves withered, stem segments showed yellowish dehydration, and plant height was significantly lower than in the other three treatments. Additionally, we observed the effects of melatonin on fresh biomass. We found that the number of new leaves, the relative water content of leaves, and the length of shoots were significantly reduced in the D group compared to the CK group. In contrast, there was a significant rising in these three indicators in melatonin-treated seedlings under drought stress (Table 1). These results indicated that melatonin application improved plant growth under drought stress.

Consistent with the change in plant morphology, there were alterations in carotenoid content in the leaves (Fig. 1B). Drought stress (D group) significantly suppressed carotenoid content compared to the CK group, suggesting that photosynthesis in the leaves was impaired. Under drought conditions, melatonin spraying (MD group) slightly upregulated carotenoid content but not significantly, implicating that the protection of *P*. *sheareri* seedlings by melatonin is not strongly dependent on carotenoid. Taken together, melatonin can protect the photosynthesis of *P*. *sheareri* seedlings from drought stress to some extent.

### Melatonin provoked activities of antioxidant enzymes

**Figure 2:**
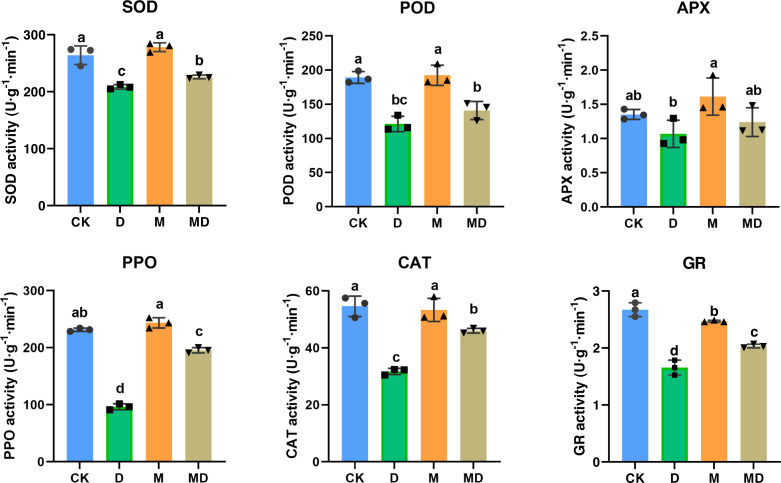
Melatonin provoked activities of antioxidantenzymes. The content of antioxidant enzymes (SOD, POD, APX, PPO, CAT, and GR) in *P*. *sheareri* seedlings in four groups.

Melatonin protected plants from oxidative damage by acting as an antioxidant enzyme activator (Imran et al., 2021). In this study, the effects of melatonin on the activity of antioxidant enzymes in *P*. *sheareri* seedlings upon drought treatment, including SOD, POD, APX, PPO, CAT, and GR, were determined (Fig. 2). The results showed that compared to the CK group, the activities of the antioxidant enzymes were significantly decreased under drought stress (D group). However, melatonin (MD group) significantly restored their activity under drought stress conditions. Notably, melatonin treatment (M group) led to a slight increase in the activities of these antioxidant enzymes under routine water treatment, compared to the CK group. Therefore, these results suggested that melatonin application could protect *P*. *sheareri* seedlings from oxidative damage induced by drought stress by provoking antioxidant enzyme activity.

**Table 1:** Melatonin treatment affects morphological indicators of P. sheareri seedlings under 2 drought stress.

**Treatment**	**Number of new leaves (*n* = 30)**	**Relative water content of leaves (*n* = 3) (%)**	**Shoot length** (*n* = 30) (cm)
CK	6.97 ± 1.30^b^	93.71 ± 1.12^a^	6.93 ± 1.39^a^
D	0.70 ± 0.74^d^	47.84 ± 3.91^c^	1.34 ± 0.58^c^
M	9.17 ± 1.79^a^	93.18 ± 1.41^a^	7.33 ± 1.38^a^
MD	4.90 ± 1.40^c^	62.14 ± 3.62^b^	2.68 ± 0.87^b^

### Melatonin restricted MDA and O_2_^•−^ accumulation

**Figure 3:**
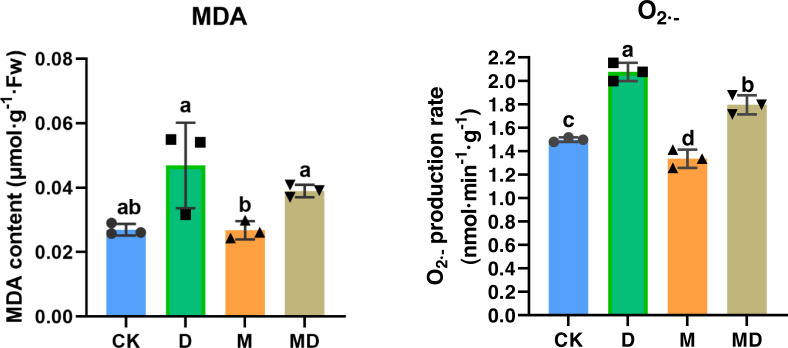
Melatonin restricted MDA and ${\mathrm{O}}_{2}^{\bullet -}$ accumulation. The content of MDA and ${\mathrm{O}}_{2}^{\bullet -}$ in *P*. *sheareri* seedlings in four groups.

Both MDA and O_2_^•−^ accumulation are indicators of impaired ROS scavenging capacity in the plants; therefore, we examined the effects of melatonin under drought stress. The results showed that drought stress (D group) resulted in a significant increase in the MDA and O_2_^•−^ accumulation in *P*. *sheareri* seedlings, compared to the CK group (Fig. 3). Expectedly, melatonin application (MD group) significantly ameliorated MDA and O_2_^•−^ accumulation induced by drought stress in *P*. *sheareri* seedlings, indicating that melatonin could function as a highly effective ROS scavenger to protect *P*. *sheareri* seedlings from drought stress.

### Melatonin affected SP, SS, and Pro contents

**Figure 4:**
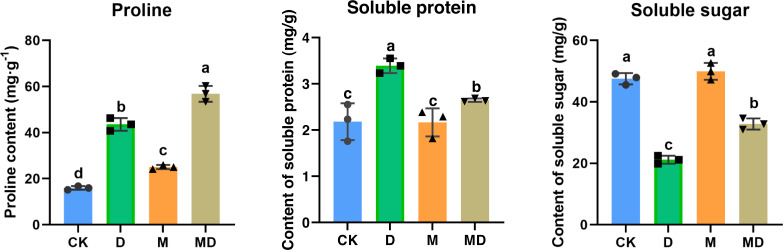
Melatonin affected SP, SS, and Pro contents. The content of proline, soluble protein, and soluble sugar in *P*. *sheareri* seedlings in four groups.

Pro, SP, and SS are all compatible solutes that increase cytoplasmic osmotic pressure, balancing water potential, and protect the membrane system, thus reducing water loss from the cells to boost the drought resistance of plants. As shown in Fig. 4, drought stress (D group) significantly increased the Pro and SP contents compared to the conventional water treatment in the CK group. It significantly reduced the SS content in *P*. *sheareri* seedlings, suggesting that its physiology was altered. In addition, under drought stress, melatonin treatment (MD group) significantly retracted SP content and increased SS content; however, melatonin treatment could not prevent the accumulation of Pro content. In conclusion, melatonin could coordinate plant resistance to drought by altering the SP and SS contents.

### Melatonin increased phytohormones content

**Figure 5:**
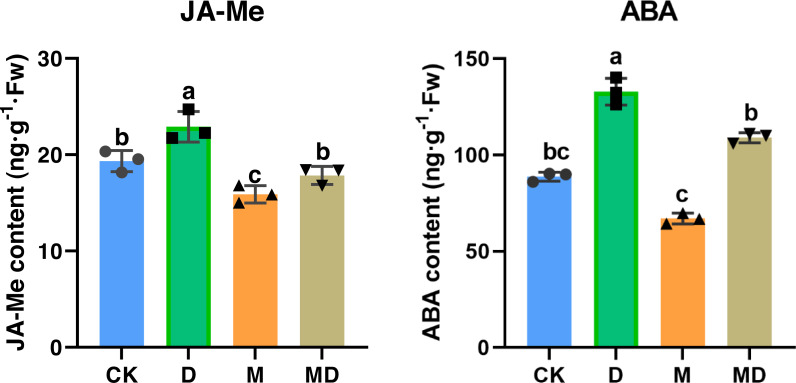
Melatonin increased phytohormone content. The content of ABA and JA-Me in *P*. *sheareri* seedlings in four groups.

We investigated whether melatonin can respond to drought stress via phytohormone regulation, such as JA-Me and ABA. The results showed that drought stress significantly increased JA-Me and ABA contents, whereas melatonin treatment significantly counteracted this alteration due to drought conditions (Fig. 5). These results suggested that melatonin enhanced the drought tolerance of *P*. *sheareri* seedlings by correcting the content of endogenous hormones, JA-Me and ABA.

### Correlation and dimensionality reduction analysis

**Figure 6:**
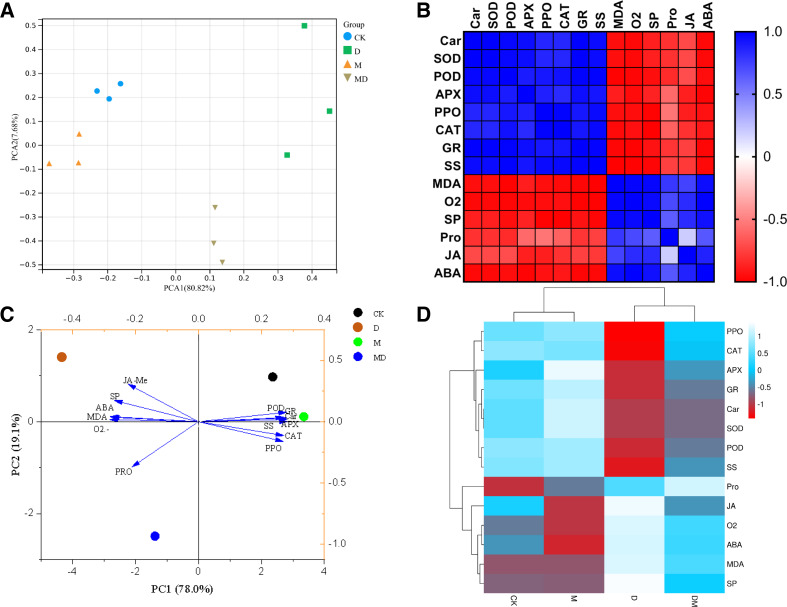
Correlation and dimensionality reduction analysis. (A) PCA analysis of correlations between samples. (B) Heatmap analysis of correlations between indicators. Blue indicates positive correlations and red indicates negative correlations. (C) PCA analysis reveals the contribution of different indicators to the grouped samples. (D) Heatmap reveals correlations between groupings and indicators, as well as clustering relationships among indicators.

To holistically evaluate the reliability of the results of this study, the samples were clustered using the PCA based on the results of all parameters. PCA reflected that the four groups have distinctly different clusters, with the CK and M groups nearby and the D group clustered away from the other three groups (Fig. 6A). The Pearson heatmap clustered a set of parameters together based on the similarity of patterns. For example, Car, SOD, POD, APX, PPO, CAT, GR, and SS were clustered into one group, and the remaining MDA, O_2_^•−^, SP, Pro, JA-Me, and ABA were clustered into one group (Fig. 6B). Moreover, the PCA of the responses of the *P*. *sheareri* seedlings to different treatments depicted 97.1% of variability in drought response (Fig. 6C). The PC1 component explained 78% of the variation and contributed positively via antioxidant enzymes (POD, APX, PPO, CAT, and GR), SS, and Car. The PC2 component explained 19.1% of the variation and contributed positively via SP, ABA, MDA, and JA-Me and negatively by O_2_^•−^ and Pro. Similarly, the comparative heatmap revealed that the antioxidant enzymes, SS, and AR are clustered together and demonstrated high levels in the CK group (Fig. 6D). The remaining parameters in this study were grouped into another cluster, including SP, ABA, MDA, O_2_^•−^, JA-Me, and Pro. According to Figs. 6B–6D, we observed that the six tested antioxidants had highly similar response patterns under drought stress and upon melatonin treatment, suggesting that it may be sufficient to measure only two or three of them from a frugality perspective.

### A comprehensive evaluation of drought resistance of *P. sheareri* seedlings under different treatment conditions

To comprehensively evaluate the effects of fourteen indicators on the drought resistance of *P*. *sheareri* seedlings, we introduced the DRC. The results showed that based on the DRC of fourteen single indicators, the comprehensive index of the MD group was the largest, followed by the D group, and the M group was the smallest (Table 2).

## Discussion

**Figure 7:**
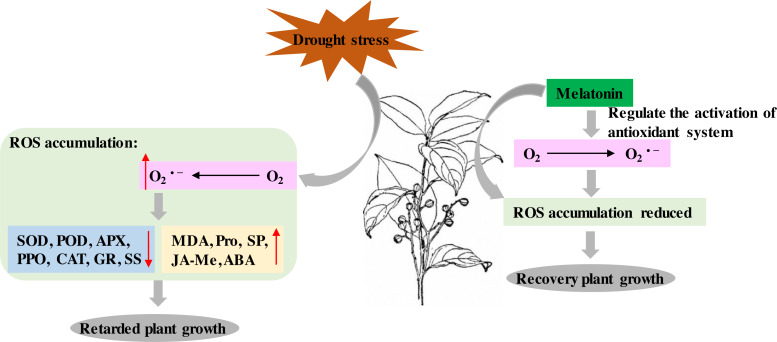
Schematic diagram of the mechanism of melatonin. The potential mechanism of melatonin to protect plants from drought stress.

*P*. *sheareri* is an endangered and National Class II protected species in China. As rainfall has decreased with global warming, the probability of *P*. *sheareri* seedlings experiencing drought stress has dramatically increased. This study revealed that spraying melatonin could resist the drought stress of *P*. *sheareri* seedlings via antioxidant defenses, cellular permeability, and endogenous hormone regulation, improving their morpho-physiological growth. To the best of our knowledge, this study is the first to demonstrate that melatonin provides resistance to *P*. *sheareri* seedlings grown under drought conditions. Based on the results and reference (Tiwari et al., 2021), we showed the potential mechanism of melatonin in protecting plants from drought stress in Fig. 7.

It is well known that drought stress leads to an inevitable accumulation of ROS, a byproduct of cellular metabolism in plants. The phytotoxic levels of ROS are detrimental, resulting in cellular damage and death (Cruz de Carvalho, 2008). However, ROS is an essential signaling molecule in plants and plays a vital role in plant resistance. ROS can trigger various stress response pathways and initiate crosstalk to regulate intracellular ROS concentrations and maintain the redox state of the cells (Verma et al., 2019). These pathways include antioxidant systems and phytohormones. In this study, drought stress dramatically increased O_2_^•−^ (common ROS) and cell membrane lipid peroxidation marker, MDA. Many studies have shown that drought stress leads to ROS stress in various plants, similar to the results of our study. For example, drought stress increased H_2_O_2_ and MDA in the *Amaranthus tricolor* cultivar (Sarker & Oba, 2018), sorghum (Nxele, Klein & Ndimba, 2017), and *Panicum sumatrense* Roth. (Ajithkumar & Panneerselvam, 2014). These results suggested that agents targeting ROS scavenging may effectively improve plant resistance to drought. This study found that melatonin treatment specifically scavenged drought-induced ROS (MDA and O_2_^•−^) accumulation, thereby improving plant growth under drought conditions. Numerous studies supported our conclusions; for example, melatonin ameliorated maize seedling’s growth by improving drought tolerance and decreasing ROS (Ahmad et al., 2021; Huang et al., 2019) as well as in cotton (*Gossypium hirsutum* L.) (Hu et al., 2021) and Tartary Buckwheat (*Fagopyrum tataricum* (L.) Gaertn) (Hossain et al., 2020). Melatonin alleviated the toxicity of nano plastics to wheat by activating the ROS scavenging system to maintain redox homeostasis and regulate the expression of aquaporin-related genes (Li et al., 2021). Melatonin could positively regulate redox homeostasis and photosynthetic carbon assimilation in barley in low-temperature conditions (Jiang et al., 2022). Therefore, the present study confirmed that melatonin enhanced the drought resistance of *P*. *sheareri* seedlings by scavenging ROS accumulation. We speculated that there were two mechanisms by which melatonin scavenged ROS. First, melatonin enhanced the expression of mitochondrial electron transport chain-related genes and accelerated the gene expression of respiratory enzymes participating in the Kreb Cycle (Turk & Genisel, 2020). Second, melatonin-mediated ascorbic acid-glutathione (AsA-GSH) scavenged ROS (Tiwari et al., 2021). In conclusion, melatonin scavenged O_2_^•−^ and restricted MDA content to mitigate oxidative damages in *P*. *sheareri* seedlings under drought stress.

**Table 2:** Comprehensive evaluation of resistance of *P. sheareri* seedlings to drought stress under different treatments.

	**Single**														**Comprehensive**
	**SOD**	**POD**	**CAT**	**Car**	**MDA**	**APX**	**PPO**	**GR**	**PRO**	**O**_**2**_^.−^	**SP**	**SS**	**JA-Me**	**ABA**	
M	1.054	1.017	0.977	1.121	0.974	1.074	1.052	0.924	1.572	0.892	0.977	1.046	0.822	0.755	1.018
D	0.789	0.64	0.581	0.621	2.109	0.704	0.416	0.619	2.731	1.386	1.417	0.443	1.184	1.497	1.081
MD	0.855	0.745	0.842	0.766	1.542	0.824	0.844	0.761	3.564	1.199	1.105	0.687	0.923	1.228	1.135

**Notes.**

Single means single index drought resistance coefficient; Comprehensive means comprehensive coefficient of drought resistance.

The present study also revealed that melatonin has an ameliorative effect on drought-induced disorders of the antioxidant enzyme system (SOD, POD, APX, PPO, CAT, and GR). Interestingly, SOD, APX, and GR are AsA-GSH cycle-related enzymes. Among them, O_2_^•−^ derived from photosystem I was scavenged by SOD which produces H_2_O_2_. Next, APX uses AsA as an electron donor to exert enzymatic translation to consume H_2_O_2_ in the cells. GR/converts reduced glutathione to oxidized glutathione and consume photosystem I-derived NAPDH (Tiwari et al., 2021). Therefore, the AsA-GSH is the mechanism by which melatonin activates antioxidant enzymes to defend against drought. Consistent with our study, the regulatory effects of melatonin on the AsA-GSH cycle in alleviating drought-induced leaf senescence in apples have been proved (Wang et al., 2013). Moreover, melatonin enhances the enzymatic antioxidants POD, PPO, and CAT activity in soybean plants (Imran et al., 2021), fenugreek (*Trigonella foenum-graecum*) (Zamani, Amiri & Ismaili, 2020), cauliflower plants (EL-Bauome et al., 2022) have been reported. Melatonin regulation may be associated with the MAPK cascade (Xia et al., 2020). Therefore, melatonin is a mediator of the different antioxidant pathways and plays a protective role in *P*. *sheareri* seedlings under drought conditions.

Melatonin can be involved in plant growth regulation as a plant growth regulator. A group systematically reviewed the mechanisms of drought stress mitigation in plants mediated by melatonin, including hormone regulation mechanisms (Tiwari et al., 2021). It has been shown that melatonin and indole-3-acetic acid (IAA) have similar molecular structures and use tryptophan as an initial synthetic substrate (Arnao & Hernandez-Ruiz, 2014). Therefore, melatonin has a similar function to IAA. In fact, melatonin has been reported to promote *Lupinus albus* growth at micromolar accumulations and inhibit growth at higher accumulations in an IAA-like manner (Arnao & Hernandez-Ruiz, 2014). In this study, we found that melatonin treatment had a down-regulatory effect on the ABA and JA-Me levels in response to drought. We speculate that this crosstalk among plant hormones may function in an IAA-like manner. Numerous studies have shown that melatonin mediated the down-regulation of ABA biosynthetic genes and up-regulation of catabolic genes under drought conditions. For example, Li et al. (2015) demonstrated that melatonin treatment reduced MdNCED3 expression of the ABA biosynthesis gene and increased ABA catabolic gene expression of MdCYP707A1 and MdCYP707A2 under water deficit conditions. However, more studies are required to prove the mechanism of melatonin regulation of JA-Me, given the conjugation of IAA and JA (Staswick, 2009). The results of this study suggested that the regulation of JA-Me by melatonin is dependent on the IAA-like mechanism. In summary, melatonin acts as a plant growth regulator of crosstalk between ABA and JA-Me to support *P*. *sheareri* seedling’s growth and withstand drought.

## Conclusions

Herein, we observed that melatonin treatment significantly improved plant growth under drought stress conditions, including promoting fresh biomass (number of new leaves, leaf water content, and shoot length), increasing the contents of Car, SOD, POD, APX, PPO, CAT, GR, and SS, and decreasing the contents of MDA, O_2_^•−^, Pro, SP, JA-Me, ABA. Moreover, the PCA analysis based on these indicators showed a clear separation between the melatonin treatment and other groups. This study demonstrated for the first time that melatonin protects *P*. *sheareri* seedlings against drought stress. This study allowed for future artificial propagation of *P*. *sheareri* seedlings and a new strategy for conserving wild *P*. *sheareri* adapted to drought.

##  Supplemental Information

10.7717/peerj.15159/supp-1 Supplemental Information 1 Raw data of fresh biomass.

10.7717/peerj.15159/supp-2 Supplemental Information 2 Raw data.

## Additional Information and Declarations

### Data Deposition

The following information was supplied regarding data availability: The raw data are available Supplemental Files.
